# Improving the catalytic activity of isopentenyl phosphate kinase through protein coevolution analysis

**DOI:** 10.1038/srep24117

**Published:** 2016-04-07

**Authors:** Ying Liu, Zhihui Yan, Xiaoyun Lu, Dongguang Xiao, Huifeng Jiang

**Affiliations:** 1 College of Biotechnology, Tianjin University of Science & Technology, Tianjin 300308, China; 2 Key Laboratory of Systems Microbial Biotechnology, Tianjin Institute of Industrial Biotechnology, Chinese Academy of Sciences, Tianjin, China

## Abstract

Protein rational design has become more and more popular for protein engineering with the advantage of biological big-data. In this study, we described a method of rational design that is able to identify desired mutants by analyzing the coevolution of protein sequence. We employed this approach to evolve an archaeal isopentenyl phosphate kinase that can convert dimethylallyl alcohol (DMA) into precursor of isoprenoids. By designing 9 point mutations, we improved the catalytic activities of IPK about 8-fold *in vitro*. After introducing the optimal mutant of IPK into engineered *E. coli* strain for β-carotenoids production, we found that β-carotenoids production exhibited 97% increase over the starting strain. The process of enzyme optimization presented here could be used to improve the catalytic activities of other enzymes.

With the availability of an increasing number of the biochemical and structural data, enzyme engineering is developing more and more from random approaches (directed evolution) to rational (data driven) design. Rational design depends on our in-depth knowledge about sequence and structure features of target proteins, which has been widely used to generate enzymes possessing desirable properties in protein engineering^1^,^2^,^3^,^4^, such as improved thermostability^5^, increased activity^6^, modified specificity^7^ and selectivity^8^. Despite of recent successes on protein design, there is no general strategy for improving activities of certain enzyme due to the complexity of the structure-function relationship.

One of the most important issues for protein rational design is how to identify responsible residues for the function of proteins. Usually, the degree of amino acids conservation is used to represent the importance in a protein^9^,^10^,^11^. However, the structure and function of a protein also depends on the cooperative interaction of amino acids^11^,^12^,^13^. Substantial effort has been dedicated to investigate the coevolutionary interaction between amino acids through statistical coupling analysis (SCA) or mutual information^14^,^15^,^16^,^17^. These co-evolving amino acids were found to be sufficient for recapitulating native folding and function of protein^15^,^16^,^17^. Furthermore, these co-evolving pairs between amino acids could contribute to identify the protein allosterism^18^,^19^,^20^ and predict the protein-protein interaction surfaces^21^,^22^. Therefore amino acids co-evolution may be a desired strategy to select residues for rational protein design.

Here, we took isopentenyl phosphate kinase (IPK) as an example to present a process of enzyme optimization based on amino acids co-evolution^23^,^24^,^25^. IPK could catalyze isopentenyl phosphate (IP) or dimethylallyl phosphate (DMAP) to produce isopentenyl diphosphate (IPP) or dimethylallyl diphosphate (DAMPP) which are the fundamental building blocks of isoprenoid compounds. Combining the structural information of IPK and functional studies^26^,^27^,^28^,^29^, we firstly found that DMA can serve as a substrate for IPK. DMA is an industrial chemical for the synthesis of pharmaceuticals and aroma compounds^30^. Therefore it provides a possible way to produce high-value isoprenoid compounds from cheap industrial chemicals. In order to improve the activity of IPK, we further implemented the analysis of protein coevolution in IPK family and designed six positions to generate mutations. Finally we examined the activity of mutants *in vitro* and *in vivo*.

## Results

### Design of a novel pathway from DMA to IPP

**Figure 1:**
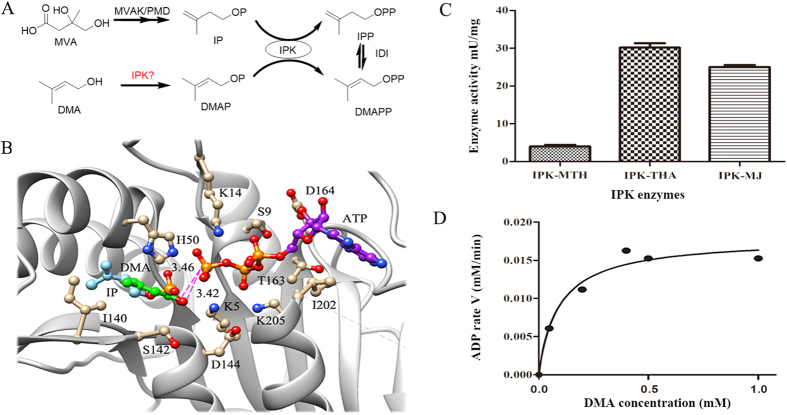
Pathways for biosynthesis of IPP and analysis of phosphorylation of DMA by IPK. (**A**) Biosynthesis of IPP, the upper one was the known pathway, the lower one was the proposed pathway (MVAK, mevalonate kinase; PMD, phosphomevalonate decarboxylase). (**B**) The structural alignment of docking poses of DMA (green) with IPK bound to substrate IP (cyan) (PDB ID 3LKK)^27^, distance between substrate and the phosphate moiety of ATP (purple) was shown as broken pink lines. (**C**) The experimental characterization for three IPK genes from different archaeal organisms, including MTH, THA and MJ. (**D**) The influence of catalytic rate for IPK gene from THA over DMA concentration.

In archaea, IPP would be produced from mevalonate (MVA)^23^,^24^,^25^ pathway, where phosphomevalonate is decarboxylated to form IP and then IP is converted into IPP by IPK (Fig. 1A). On the other hand, DMAP can also be phosphorylated by IPK to produce DMAPP^25^. At the same time, the recombinant of IPKs from *E. coli* and peppermint performed the ability of converting DMA to DMAP, but DMAP did not serve as a substrate^29^. These studies prompted a possible pathway from DMA to IPP, where DMA could be phosphorylated by IPK twice and then DMAPP could become IPP by IDI (Isopentenyl diphosphate isomerase)^31^, as shown in Fig. 1A. Indeed, the molecular docking analysis indicated that DMA was a potential substrate for the archaeal IPK (Fig. 1B). When IPK bound to DMA and ATP at the same time, the distance of a nucleophilic oxygen atom of the OH group in DMA was 3.42 Å from the electrophilic P_γ_ phosphate atom in ATP, which was similar to the crystal structure of IPK with the substrate IP^26^,^27^,^28^. Therefore, we proposed a novel pathway from DMA to IPP via IPK and IDI genes.

To examine the possibility of phosphorylation of DMA by IPK, three IPK genes from *Methanothermobacter thermautotrophicus* (MTH), *Thermoplasma acidophilum* (THA), *Methanocaldococcus jannaschii* (MJ), were overexpressed and proteins were purified (Supplementary Fig. 1), respectively. The phosphorylation activity was measured by monitoring the PPi release rate, where the degree of ATP consumption reflected the turnover rate of the enzymatic reaction^32^. The result showed that DMA can be regarded as a substrate for three IPKs (Fig. 1C). Although the activity of DMA phosphorylation by IPK was not high enough (the highest one from THA is *K*_*m*_ = 0.0945 (mM), *k*_*cat*_ = 3.13 (min^−1^)) (Fig. 1D), IPP can be synthesized by the alternative pathway from DMA.

### Coevolution analysis of IPK

Protein co-evolution has been used to identify important residues for protein folding and function^15^,^16^,^17^, which promoted us to perform coevolutionary analysis for IPK gene. Firstly we obtained 45 orthologous genes of IPK from eggnog database^33^ (Supplementary Fig. 2). To further detect more homologous genes of IPK, we constructed a hidden Markov model (HMM)^34^ for IPK family using these orthologous genes and performed HMM search in NCBI non-redundant database. As a result, 483 putative homologous genes for IPK gene family were identified. After removing gaps from multiple sequence alignment among these genes, 245 amino acids have been retained for each gene. Subsequently, these aligned sequences were used for the coevolution analysis by SCA method^11^,^12^ (Methods).

**Figure 2:**
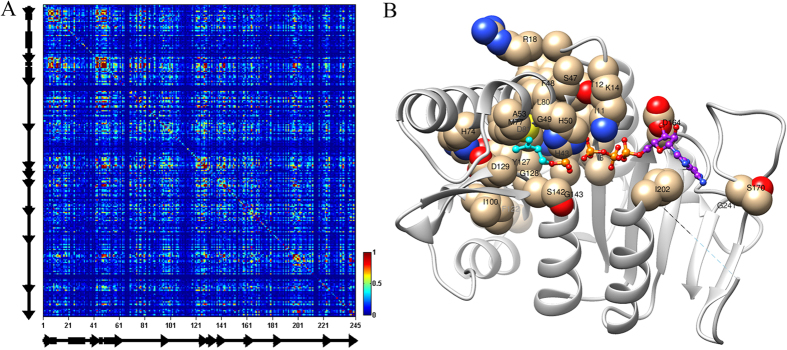
The coevolving analysis of IPK family protein. (**A**) SCA matrix for a sequence alignment of 483 members of the IPK family. (**B**) Highly coevolving positions (positions of interaction number with more than 10) were mapped into IPK structure.

Based on coevolution analysis, in fact many residues had significant correlation not only with neighboring residues, but also with residues that were distant along the sequence (Fig. 2A). However, from the protein structure, we found that those strongly co-evolved residues prefer to be embedded into the catalytic center (Fig. 2B). For example 29 out of 245 positions co-evolved with more than 10 other positions (Supplementary Fig. 3). 20 of them were located within 10 Å distance from the substrates and 7 positions distributed at the ATP binding region (D164, S170, D197, V198, G201, I202, G241) and the rest two positions were on the interaction surface of IPK dimerization (R18 and F122, see PDB ID 3LKK)^27^. Therefore, these positions would be important for maintaining the structure and function of IPK.

### Improving the activities of IPK *in vitro*

In order to improve the catalytic activities of IPK, we avoided to mutate residue at the position which has strong coevolutionary interaction with other positions as far as possible. Among 19 residues that either directly interact with the substrates (within 5 Å distance from the substrates) or involve in the substrate channel, 9 positions (H43, G49, H50, A53, M77, G128, D129, S142, G143) strongly coevolved with others (Supplementary Fig. 3). At the same time, 4 of them (K5, G44, G46, D144) were very conserved (Supplementary Fig. 4). Therefore, the rest 6 residues (G45, V73, V130, I140, Y141, K204) were possible mutated targets which could not destroy the function of IPK.

**Figure 3:**
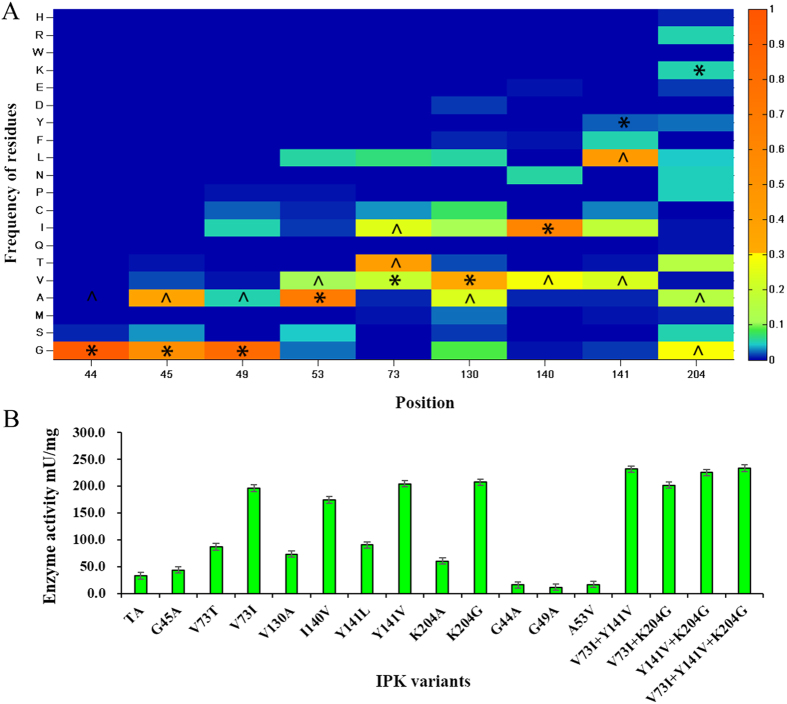
Mutant design and the experimental result. (**A**) The distribution of frequency of occurrence of amino acid for mutated targets and controls. *stands for the WT in each position, ^stands for mutants. (**B**) The enzyme activities. G44, G49 and A53 were selected as controls.

As we known, natural selection prefers to maintain amino acids which have positive impact on protein function^35^,^36^. The higher occurrence of frequency of amino acid is, the better the amino acid at the position is. Therefore we mutated amino acids at these sites into the amino acids which have high frequency at these sites in IPK family (Fig. 3A). Meanwhile, we also selected amino acids from one conserved site (G44) and 2 strongly coevolved sites (G49 and A53) as controls (Supplementary Fig. 4A).

Based on these analysis, we carried out experiments on a set of designed mutants. The experimental results showed that the designed mutants for possible mutated targets indeed can improve the activities of IPK (Fig. 3B). Moreover among these mutants, V73I, Y141V and K204G exhibited about 7-fold activity increase for the substrate of DMA. In contrast these conserved and strongly coevolved sites (G44, G49 and A53) does result in decrease of catalytic activity. To further improve the catalytic activity of IPK, the three mutants with higher activity were combined together. As a result, the combined mutant exhibited about 8-fold activity increase compared to the original IPK (Fig. 3B).

### Raising DMAPP production *in vivo*

IPP and DMAPP are two common building blocks for the synthesis of isoprenoids^37^,^38^,^39^,^40^. In order to evaluate the effect of phosphorylation of DMA by IPK *in vivo*, here we took the isoprenoid compound β-carotene as an example. An engineered *E.coli* QL105 strain could use glucose as resource to produce β-carotene via methylerythritol phosphate (MEP) pathway^41^. If IPK gene is transformed into the strain, DMA will be one of resources to synthesize β-carotenoids (Supplementary Fig. 5). Thus comparing with QL105, the increase of β-carotenoids yield would be resulted from the transformation of DMA by IPK. The experimental results (Table 1) showed that the yield of β-carotene increased 0.87 mg/g DCW (Dry cell weight) when the recombinant plasmid with IPK gene was transformed into *E.coli* QL105 strain. When the highest activity mutant of IPK gene (V72I + Y140V + K203G) was transformed into QL105 strain, the yield of β-carotene increased 1.31 mg/g DCW. In order to further promote the transformation from DMA to β-carotene, the isomerase *idi* gene that convert DMAPP into IPP was overexpressed and the yield of β-carotene increased 1.86 mg/g DCW. Therefore after introducing the mutant of IPK into *E. coli*, the yield of isoprenoid β-carotene increased about 1-fold, suggesting that IPK indeed can convert DMA into DMAPP and increase the production of β-carotene.

## Discussion

Isoprenoids contain more than 30,000 compounds, which is the largest family of natural products in the world^42^. IPP and DMAPP are the fundamental five-carbon building blocks of isoprenoid compounds in all organisms. Different from the natural mevalonate (MVA) and methylerythritol phosphate (MEP) pathways, here we proposed an alternative pathway for synthetizing DMAPP and other isoprenoids from an industrial chemical compound DMA. The substrate DMA could be phosphorylated twice by IPK to generate DMAPP. Taking an example, DMAPP further could be converted into other isoprenoids like β-carotene. Although the yield of β-carotene in our study was lower than the highest production (72 mg/g)^43^, our results not only established an unnatural pathway for isoprenoids synthesis and also provided a possible way for producing high-value isoprenoids from cheap industrial chemical compound.

To improve the catalytic activity of IPK, we firstly implemented protein co-evolution analysis to detect key sites for mutation. Although the catalytic activity of IPK was only improved about 8-fold, it was very successful when we conducted point mutations. All of designed mutants based on evolutionary analysis increased the enzyme activities. Interestingly some mutants might change the catalytic pocket in the enzyme. For example, K204 involves in substrate channel and I140 is located on surface of pocket. Therefore the substitution of both positions with smaller residues maybe facilitate the access for substrate or product (Supplementary Fig. 6). Surprisingly, it seems that most of DMA had been converted into the product β-carotene because eight DMA molecules will constitute one β-carotene and about 8-fold increase of DMA will result in one-fold increase of β-carotene. Therefore the improvement of IPK activities in future would further increase the production of β-carotene.

In summary, we presented an example of functional detection of IPK for substrate DMA and improving IPK activities by protein co-evolution analysis. *In vitro*, the activity of optimal mutant has been increased to ~8-fold compared to WT. *In vivo*, most of substrate DMA had been transformed into β-carotene and the yield increased about one-fold over the parent strain QL105. Therefore, our results not only provided a novel pathway for synthesizing isoprenoids, but also proposed a strategy of rational protein design.

## Methods

### Molecular docking

In order to detect the interactions of DMA in the active site of archaeal IPK, the Autodock4.2 program^44^ was used to perform the molecular docking studies. The crystal structure of IPK in complex with substrate IP (PDB entry 3LKK)^27^ was abstracted from PDB, and was treated manually as follows; all water molecules found in structure were stripped, and the hydrogen bonding networks were added. For docking protocol, the enclosing box was centered on substrate IP, and the spacing was set to 0.4 Å, the other parameters were default.

### Sequence alignment construction

Homologous sequences comprising the IPK family were collected from the NCBI non-redundant database through search of IPK motif. HMMER software^33^ was used to construct the IPK motif, these initial query sequences from archaeal organisms and alignment was displayed in Supporting Information (Supplementary Fig. 2). Muscle^45^ was used to perform the multiple sequence alignments (MSA) of homologous sequences of the IPK family. To improve the quality of subsequent sequence analysis, the sequence positions with gap was deleted based on the target sequence (IPK from THA) with the help of trimAl program^46^,^47^. The final alignment consisted of 483 sequences and 245 amino acid positions.

### Statistical coupling analysis

One of the earliest and most popular methods, Statistical Coupling Analysis (SCA), was used to measure the conservation and pairwise correlation in the multiple sequence alignment and was first described in Lockless and Ranganathan^11^,^12^. In brief, SCA gives a matrix that calculates the weighted sequence correlation between any two positions and a positional conservation based on the background frequency of amino acid. The SCA programs can be obtained online from http://systems.swmed.edu/rr_lab/sca. html, and a detailed procedure for the SCA calculation was described previously by Halabi *et al*.^11^. To clearly distinguish the tendency of co-evolving network, a higher correlation coefficient was selected as a cutoff value to analyze the tendency of co-evolving network of each position. In paper, we chose 0.75 as cutoff value (0.8 or 0.9 also was working).

### Cloning and *in vitro* mutagenesis

The IPK genes from THA, MTH and MJ were optimized to the codon usage of *E.coli* and synthesized, respectively. These synthetic genes were ligated into expression vector pET-28a via *Nde*І and *Xho*I restriction sites to construct the IPK plasmids (pET-28a-THA, pET-28a-MTH, pET-28a-MJ). Subsequently, these plasmids were cloned into the expression vector *E.coli* BL21 (DE3) strain (Novagen, USA), respectively. In aspect of mutations, the Fast Site-directed Mutagenesis kit (Transgen, China) was used to introduce mutations based on the pET-28a-THA plasmid.

### Expression and purification of IPK in *E. coli*

Cells expression of wild-type and variants was grown to an OD_600_ of 0.6 at 37 °C in Luria-Berani medium (1% tryptone, 0.5% yeast extract, 1% NaCl) supplemented with 50 μg/ml kanamycin, and then induced at 16 °C overnight by the addition of isopropyl-β-D-thiogalactopyranoside (IPTG) to a final concentration of 0.5 mM. The wild-type and mutant IPK enzyme were harvested by centrifugation at 5500 rpm for 30 min and stored at −80 °C until needed.

Frozen cell paste was thawed on ice and suspended in 35 ml lysis buffer (100 mM Tris-HCl, 200 mM NaCl, pH 7.5). The cells were disrupted by a high-pressure homogenizer (JNBIO, China) on ice, and the cell debris was removed by centrifugation at 15000 rpm for 30 min. To bind the recombinant enzyme, which was expressed as a fusion protein containing a 6-His tag, the supernatant was filtered and loaded onto a Ni^2+^-chelating affinity chromatography column (GE Healthcare, USA). After the column was rinsed with 50 ml wash buffer (100 mM Tris-HCl, pH 7.5, 200 mM NaCl and 50 mM imidazole), the proteins were eluted with 20 ml elution buffer (100 mM Tris-HCl, pH 7.5, 200 mM NaCl and 200 mM imidazole). The eluted proteins was concentrated and dialyzed against lysis buffer (100 mM Tris-HCl, 200 mM NaCl, pH 7.5) by ultrafiltration with an Amicon Ultra centrifugal filter device (Millipore, USA) with a 30 kDa molecular-weight cutoff. The purity of the protein IPK was evaluated by 12% SDS-PAGE. Protein concentration was determined by BCA Protein Assay Reagent Kit (Pierce, USA) with BSA as a standard.

### IPK activity assay and kinetic properties

All specific activity and kinetic measurements were performed by measuring the generation of ADP through a coupled assay with pyruvate kinase and lactate dehydrogenase^32^. An initial continuous assay runs at room temperature included 7 U pyruvate kinase, 10 U lactate dehydrogenase, 2 mM PLP, 0.16 mM NADH, 50 mM Tris-HCl, pH 8.0, 100 mM KCl, 8 mM MgCl_2_, 2mM ATP and 0.5 mM DMA in a final volume of 200 μl. The reaction was initiated by the addition of IPK (0.3 μg) and then an initial linear decrease in absorbance at 340 nm, expressed as ∆(AU340)/∆t and converted to ∆(ADP)/∆t, was observed. One unit of enzyme activity was defined as the amount of enzyme that catalyzes the conversion of 1 μmol of ATP into ADP per minute. For kinetic assays, the concentration of DMA was adjusted from 0 mM to 2 mM. Kinetic parameters *k*_*cat*_ and *K*_*m*_ were estimated from the slope and intercepts of the Lineweaver-Burk plot using Microsoft Excel software.

### Analyzing product of enzymatically generated reaction in *E.coli* QL105 strain

In order to further confirm that the production of DMAPP, IPK gene from THA and mutant with the highest activity were transformed into engineering strain *E.coli* QL105 (QL105 strain integrated the β-carotene synthetic *crt* gene and the *crt* gene operon together with *trc* promoter and *rrnB* transcriptional terminator)^41^. At the same time, *idi* gene that was responsible for the isomerization of the carbon-carbon double bond of DMAPP to produce IPP was overexpressed in strain with mutant. DMAPP and IPP are two important precursors for synthetizing β-carotene. Therefore, the effect of phosphorylation of DMA can be reflected by monitoring the yield of β-carotene.

Electro-competent *E.coli* QL105 cells were prepared as described^41^, and used as a host strain for transformation of recombinant plasmid pTrc99A-IPK-THA. Electro-competent cells (40 μl) were electroporated with 0.5 μg recombinant plasmid using a Bio-Rad Gene Pulser set at 1.8 kV. Shocked cells were added to 1 ml SOC medium (Invitrogen, Carlsbad, CA), incubated at 37 °C for 2 h and then spread on Luria-Bertani (LB) plates containing both 25 μg/ml Ampicillin overnight to select antibiotic-resistant recombinants.

Yield of β-carotene was determined by measuring the absorption of the acetone-extracted β-carotene at 453 nm as described previously^48^ and then normalizing it to the cell density (OD_600 _nm). The *E.coli* QL105 strain harboring a recombinant plasmid with IPK and *idi* gene was grown in 5 ml Luria-Berani medium containing 50 μg/ml Ampicillin and 2 mM DMA at 37 °C until OD_600_ reached 0.6–0.8. Expression of the recombinant protein IPK and IDI was induced by adding 1 mM IPTG and growth of the culture continued at 30 °C overnight. Cells were harvested by centrifugation at 5500 rpm for 15 min. β-carotene pigments were extracted from the cell pellet by resuspending in acetone (1 ml) and incubated at 55 °C for 15 min in dark. After centrifuging the sample at 14000 rpm for 10 min, the absorption spectrum of the acetone supernatant containing β-carotene was measured by using a Shimadzu UV-2550 spectrophotometer (Shimadzu, Kyoto, Japan). A standard curve (Supplementary Fig. 7) was obtained by measuring OD_453_ of serially diluted β-carotene standard samples (Cat. No. C4582, Sigma, USA) and then used to calculate β-carotene production of each strain. Dry cell weight (DCW) was calculated from the optical density at 600 nm (1OD_600_ = 0.323 g DCW/L).

## Additional Information

**How to cite this article**: Liu, Y. *et al*. Improving the catalytic activity of isopentenyl phosphate kinase through protein coevolution analysis. *Sci. Rep.* **6**, 24117; doi: 10.1038/srep24117 (2016).

## Supplementary Material

Supplementary Information

## Figures and Tables

**Table 1:** Monitoring of β-carotene production in strain QL105 with IPK and IPK variant.

Strain^a^	OD_600_	Dry cell weight (g/L)	OD_453_^b^	β-Carotene yield (mg/g)
QL105	4.68 ± 0.00	1.51 ± 0.00	0.35 ± 0.00	1.92 ± 0.00
IPK-THA	4.81 ± 0.01	1.55 ± 0.01	0.46 ± 0.00	2.79 ± 0.00
V72I + Y140V + K203G	4.66 ± 0.11	1.50 ± 0.11	0.51 ± 0.03	3.23 ± 0.00
V72I + Y140V + K203G + IDI	4.54 ± 0.05	1.47 ± 0.05	0.67 ± 0.01	3.78 ± 0.01

^a^The experiment was repeated three times for each strain, and the error bars represented standard deviation.

^b^Acetone-extracted β-carotene solution was concentrated 2 times for measuring the absorption at 453 nm.
