# Supplementary material for: Improving the catalytic activity of isopentenyl phosphate kinase through protein coevolution analysis
Source: Sci Rep. 2016 Apr 7;6:24117. doi: 10.1038/srep24117 (PMC4823809; doi:10.1038/srep24117)
Supplement: Supplementary Information [file srep24117-s1.pdf]

Improving the catalytic activity of isopentenyl phosphate kinase through protein coevolution analysis

Ying Liu[1,2,*], Zhihui Yan[2,*], Xiaoyun Lu[2], Dongguang Xiao[1], Huifeng Jiang[2]

[1]College of Biotechnology, Tianjin University of Science & Technology, Tianjin 300308, China

[2]Key Laboratory of Systems Microbial Biotechnology, Tianjin Institute of Industrial Biotechnology, Chinese Academy of Sciences, Tianjin, China

* These authors contributed equally to this work.

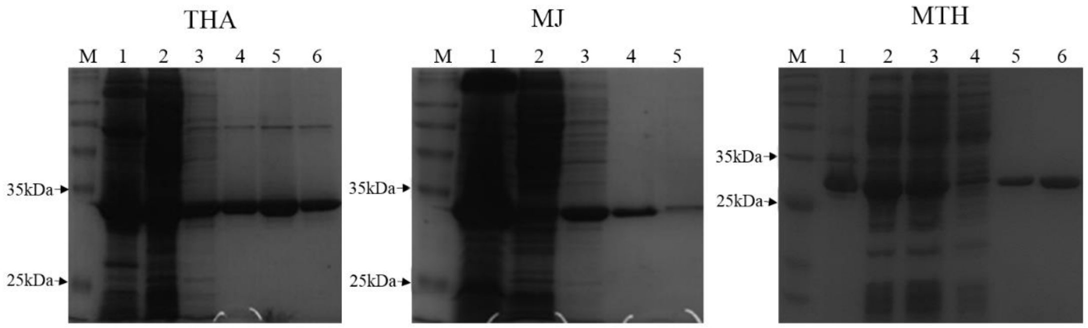

Supplementary Figure 1. Purification of IPK as shown by SDS-PAGE. M, molecular mass markers; Lane1, precipitation samples in the cell lysates; Lane2, supernatant samples in the cell; Lane3, 4, 5 and 6, purified IPK from 50, 100, 200 and 300 mM imidazole gradient elution, respectively.

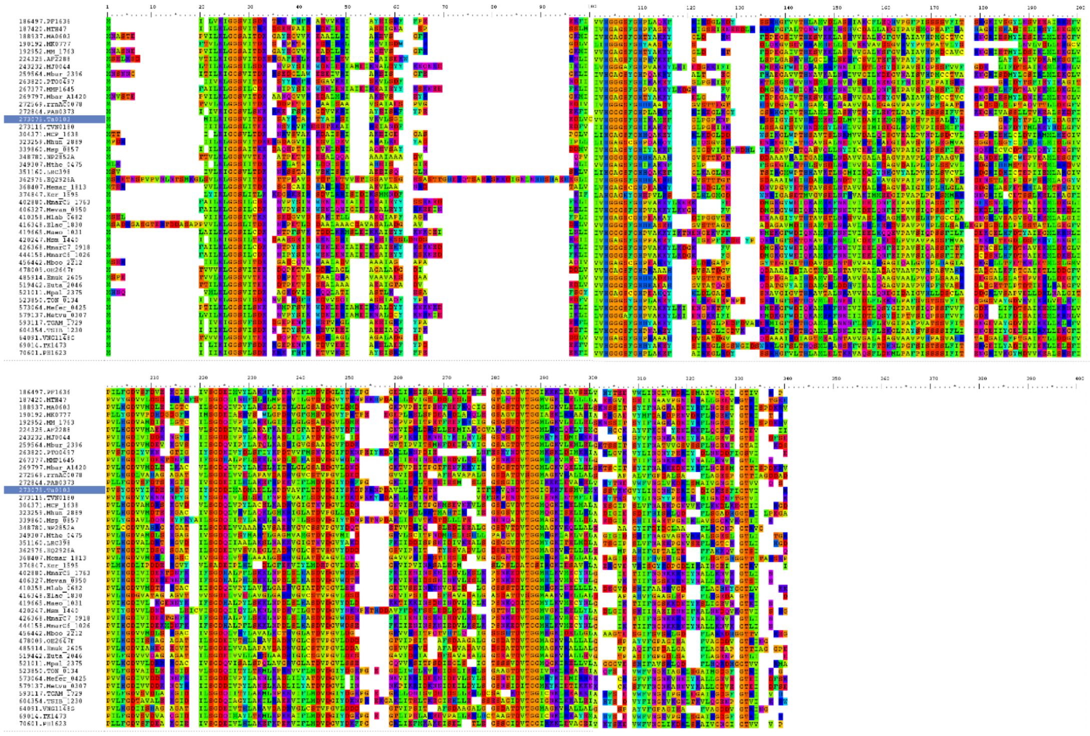

Supplementary Figure 2. The orthologous genes (eurNOG02481) of IPK family from archaeal organisms and alignment, the number of left side stands for species, the string stands for gene name, item with blue color is sequence of target protein IPK. The sequences and the aligned file can be obtained from EggNog database (http://eggnog.embl.de/version_3.0/downloads.html). eurNOG02481 is the serial number of euryarchaeota non-supervised orthologous groups (eurNOG) for IPK in the database.

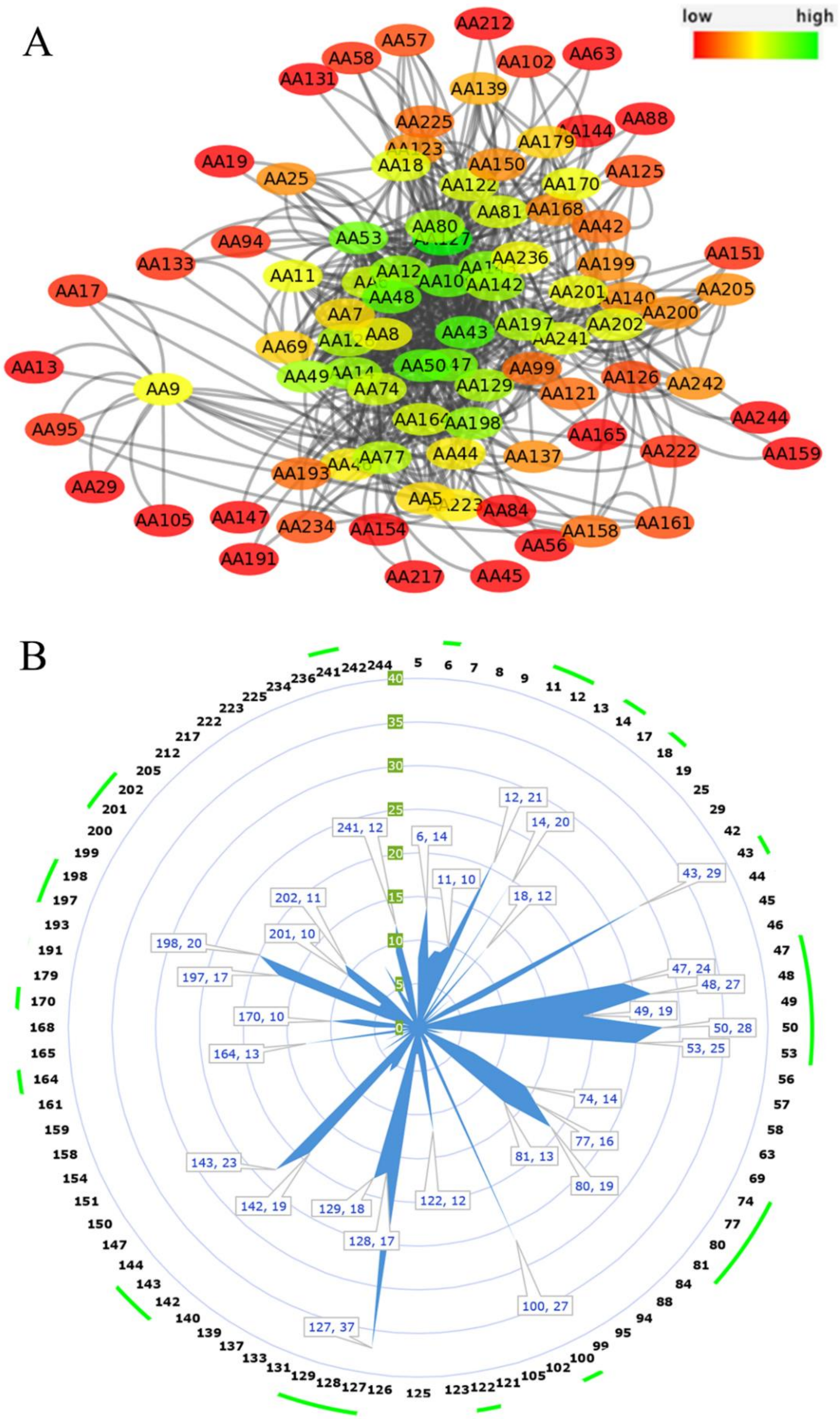

Supplementary Figure 3. Analysis of interaction network of the coevolved pairs. (A) Co-evolved network of IPK when the cutoff of correlation was 0.75. The positions that have stronger coevolutionary interaction with other positions tend to be green, the positions that have weaker coevolutionary interaction with other positions tend to be red. (B) The data tagging of coevolving pairs with more than 10 was shown.

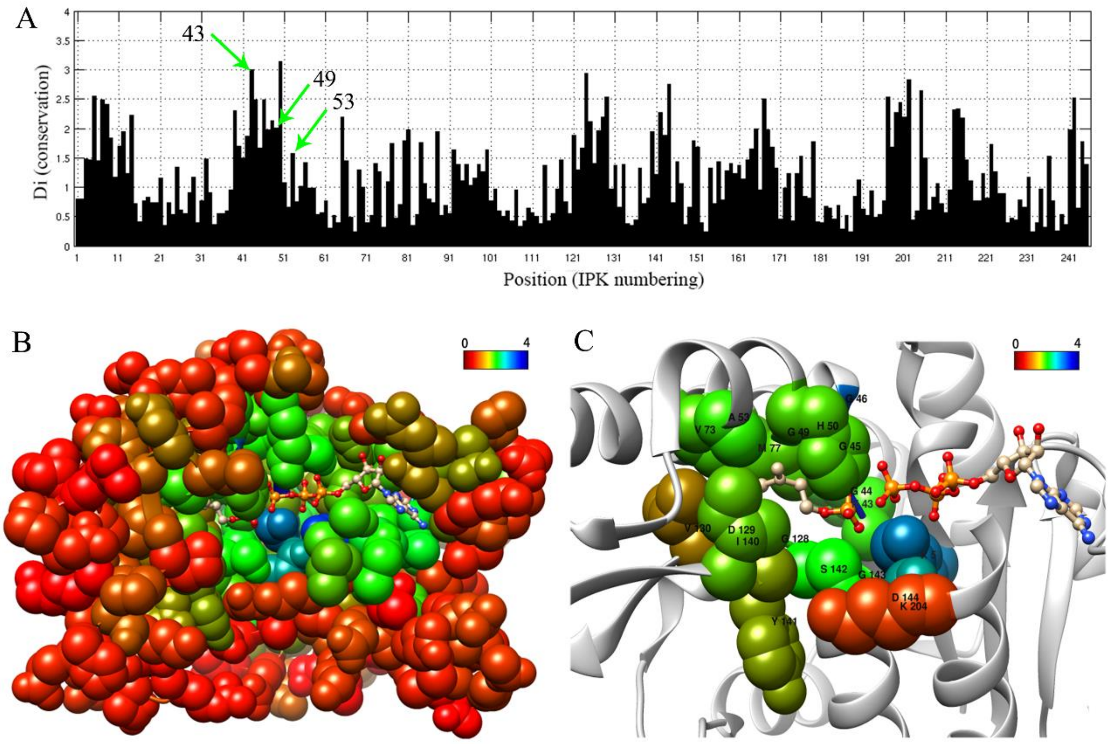

Supplementary Figure 4. The conservative analysis of IPK. (A) Conservation of IPK, the green arrows show the conservation of residues in controls. (B) Mapping of the moderate to strongly conserved positions in a surface view. (C) Mapping the conservation into functional region of IPK from THA. The conservation of each position in a multiple sequence alignment of 483 members of the IPK family, computed by the relative entropy D. More conserved positions tend to be blue, less conserved positions tend to be red.

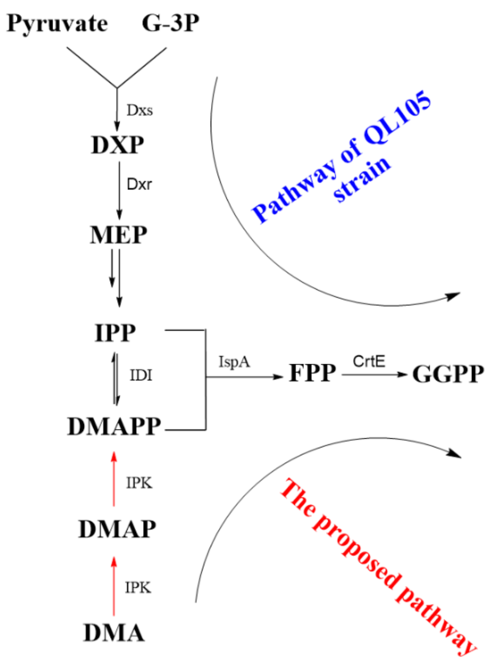

Supplementary Figure 5. Synthetic pathway of β-carotene using DMA as substrate in strain QL105. Abbreviation: G-3-P, glyceraldehyde-3-phosphate; DXP, 1-deoxy-D-xylulose-5-phosphate; MEP, 2*C*-methyl-D-erythritol-4-phosphate; DMA, dimethylallyl alcohol; DMAP, dimethylallyl phosphate; DMAPP, dimethylallyl diphosphate; IPP, isopentenyl diphosphate; FPP, farnesyl diphosphate; GGPP, geranylgeranyl diphosphate.

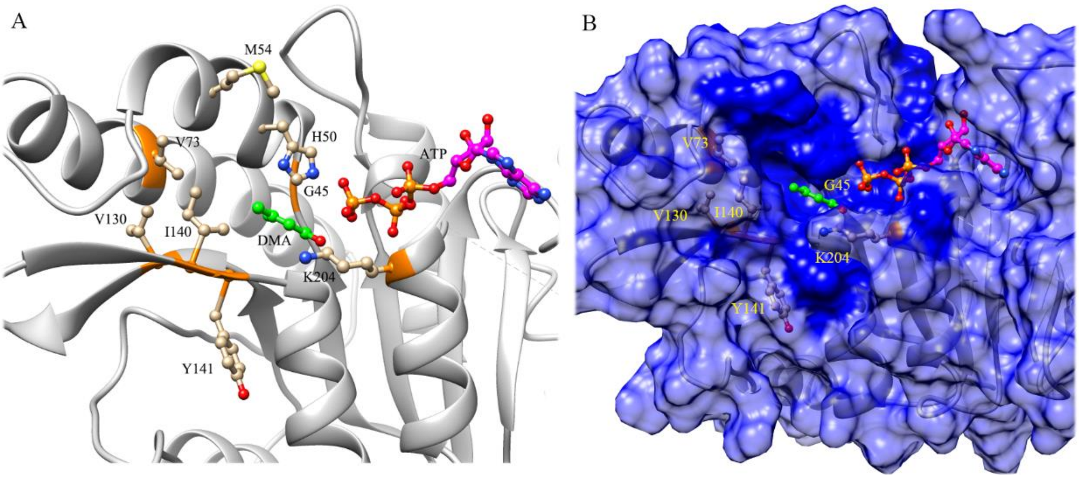

Supplementary Figure 6. Positions of the improved mutant (orange) were mapped into IPK model with substrate DMA (green) and cofactor ATP (purple). A represented ribbon diagram of IPK structure, B represented surface of IPK structure.

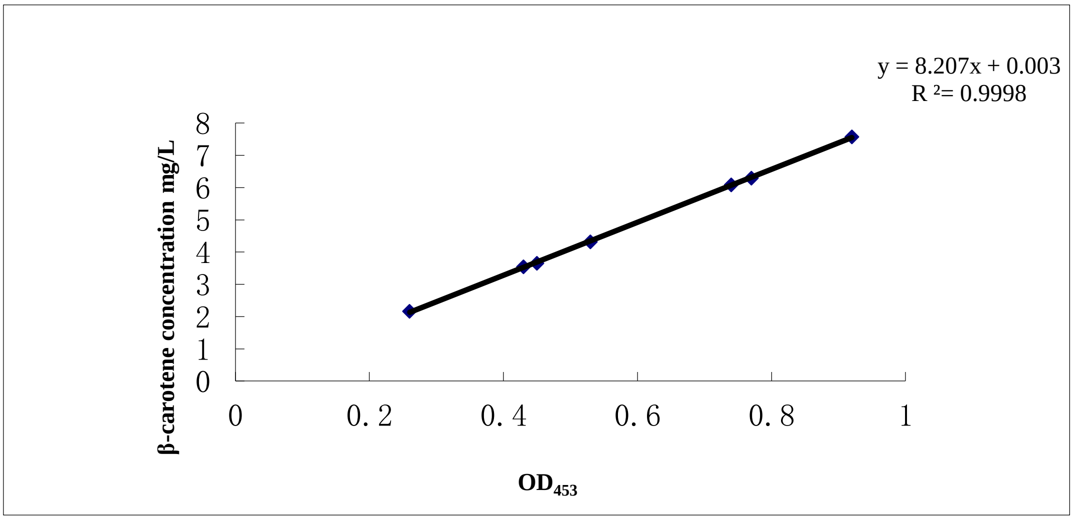

Supplementary Figure 7. Standard curve for calculating β-carotene concentration through the absorption of the acetone-extracted β-carotene at 453 nm.
